# Effects of Prophylaxis with Oral Supportive Care for Peri-implantitis in Patients Undergoing Malignancy Chemotherapy

**DOI:** 10.3290/j.ohpd.b2183011

**Published:** 2021-10-22

**Authors:** Yoshimi Ohnishi, Takashi Fujii, Jun Ishikawa, Miki Ishibashi, Masahiko Higashiyama, Shin-ichiro Hiraoka

**Affiliations:** a Dental Hygienist, Department of Dentistry, Osaka International Cancer Institute, Osaka, Japan. Wrote the manuscript, read and approved the final manuscript.; b Head and Neck Surgeon, Department of Head and Neck Surgery, Osaka International Cancer Institute, Osaka, Japan. Participated in administering the questionnaire, read and approved the final manuscript.; c Hematologist and Oncologist, Department of Hematology and Oncology, Osaka International Cancer Institute, Osaka, Japan. Participated in administering the questionnaire, read and approved the final manuscript.; d Dentist, Department of Dentistry, Osaka International Cancer Institute, Osaka, Japan. Participated in administering the questionnaire, read and approved the final manuscript.; e Thoracic Surgeon, Department of Dentistry, Osaka International Cancer Institute, Osaka, Japan; Department of General Thoracic Surgery, Osaka International Cancer Institute, Osaka, Japan. Critically revised the manuscript, read and approved the final manuscript.; f Oral and Maxillofacial Surgeon and Assistant Professor, First Department of Oral and Maxillofacial Surgery, Graduate School of Dentistry, Osaka University, Osaka, Japan. Designed the study, helped in drafting the manuscript, read and approved the final manuscript.

**Keywords:** cancer, chemotherapy, febrile neutropenia, oral supportive care, peri-implantitis

## Abstract

**Purpose:**

Dental implants without proper maintenance may lead to serious consequences, such as peri-implantitis. Peri-implantitis in patients undergoing antitumour chemotherapy can negatively affect the prognosis of treatment. The purpose of this study was to examine the association between the onset of peri-implantitis and the effects of oral hygiene management in patients with dental implants undergoing antitumour chemotherapy.

**Materials and Methods:**

Twenty-three patients (n = 23) with dental implants who received oral supportive care during malignancy chemotherapy were included. They were categorised into two groups based on the presence of peri-implantitis and were analysed for oral hygiene conditions, maintenance after implant insertion, and adverse effects such as febrile neutropenia. Statistical analysis was performed using the Fisher’s exact test and the Mann-Whitney U-test, with p < 0.05 considered statistically significant.

**Results:**

The average number of implants was higher in patients with peri-implantitis; these implants generally did not receive appropriate maintenance. There were statistically significantly fewer peri-implantitis sites in patients receiving continuous implant maintenance therapy than those who did not (p < 0.05). The severity of febrile neutropenia was reduced by dental interventions.

**Conclusion:**

Dental intervention before malignancy chemotherapy effectively prevented peri-implantitis and contributed to alleviating febrile neutropenia, even when it was initiated amidst chemotherapy. Dental intervention before chemotherapy seems essential in malignancy patients with dental implants.

Dental implant therapy has been widely performed in recent years. The prevalence of peri-implantitis increases in patients who do not receive appropriate maintenance after treatment.^6,7,21^ The incidence of peri-implantitis has been reported to be 28% at 5 years after implant insertion and ranges from 28% to 56% over 5 to 20 years after implant insertion.^6,7,21^ According to one report, the incidence of peri-implantitis was 77.4% among patients in whom an implant specialist completed dental implant treatment and who subsequently received maintenance treatment in a general dentistry practice.^17^ In addition, according to a large meta-analysis,^9^ the prevalence of implant-based peri-implantitis was 9.25% (95% CI: 7.57, 10.93), and the subject-based prevalence was 19.83% (95% CI: 15.38, 24.27).^9^

However, there are few reports on the actual onset of peri-implantitis among a patient cohort large enough to provide statistically significant results or draw meaningful conclusions. In addition, no study has been performed among malignancy patients with peri-implantitis, and the only literature available consists of a case report on the use of a bone-modifying agent for cancer therapy, alongside another case report on dental implant treatment after surgery for cancer.^11,19^

The onset of peri-implantitis during antitumour chemotherapy may result in severe complications, such as febrile neutropenia, which may lead to the discontinuation of certain treatments or reduction in drug dosages. This, in turn, can affect the patient’s quality of life and vital prognosis.

Supportive treatment after implant therapy is essential.^8,13,17^ In malignancy patients, the incidence of peri-implantitis may be higher due to discontinued consultation with dentists, in addition to decreased physical strength and immunity associated with the disease and its treatment.

This study examined the association between the onset of peri-implantitis and the effects of oral hygiene management in patients with dental implants undergoing antitumour chemotherapy.

## Materials and Methods

### Study Period and Subjects

This study included patients with dental implants consisting of pure titanium screw-type implant fixtures, who underwent antitumour chemotherapy at our institution between April 2014 and September 2016. All investigations were performed according to protocols that were reviewed and approved by the appropriate ethics committee. The study was conducted in accordance with the 1964 Declaration of Helsinki, and informed consent was not required because of the retrospective study design.

### Methods

A retrospective review of the patients’ medical records was performed to retrieve the following information: implant-related information (number and sites of dental implants, number of years elapsed since implant insertion, presence or absence of history of peri-implantitis, and time of onset of peri-implantitis); oral environment-related information (number of remaining natural teeth, oral hygiene status, initiation of medical care in our department, presence or absence of routine oral hygiene maintenance until the initiation of medical care in our department); information on malignancy therapy and systemic conditions (type of malignancy and malignancy treatment regimen); medical history, medication history, and smoking history; presence or absence of myelosuppression and its severity; white blood cell and neutrophil counts; treatment-related complications including the presence or absence of febrile neutropenia. All the criteria were defined as per the National Institute of Health’s Common Terminology Criteria for Adverse Events (CTCAE).^20^

Good oral hygiene status was defined as a plaque control record value < 20% at the initial diagnosis in our department. Peri-implantitis was defined as redness and swelling of the peri-implant mucosa, presence of draining sinuses, and/or obvious radiographic findings of peri-implant bone resorption. The depth of peri-implant pockets was not measured because bleeding on probing is a risk factor of uncontrolled bleeding in patients with haematological malignancies.

### Statistical Analysis

The methodology for this study was reviewed by an independent statistician. Continuous variables are expressed as mean ± standard deviation, and categorical variables are expressed as numbers and percentages. Data on oral hygiene condition and maintenance after implant insertion were compared between patients with and without peri-implantitis using Fisher’s exact test. The difference in the onset of febrile neutropenia between groups was analysed using the Mann-Whitney U-test. Statistical significance was set at p < 0.05, and all statistical analyses were performed using IBM SPSS Advanced Statistics 20.0 (Armonk, NY, USA).

## Results

**Fig 1:**
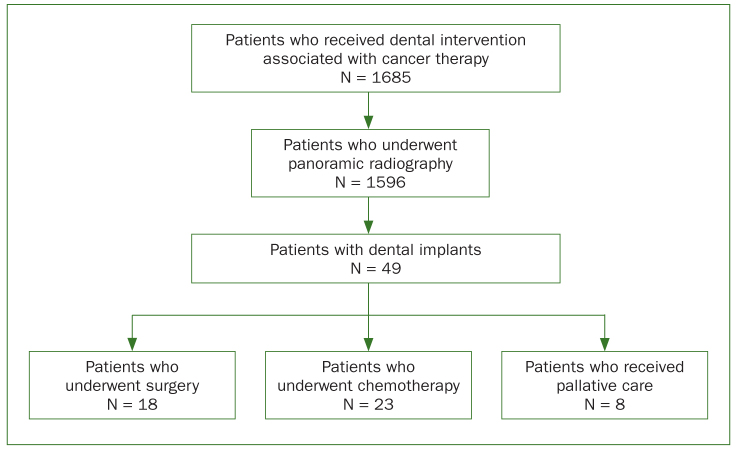
Selection process and general characteristics of study subjects.

**Fig 2:**
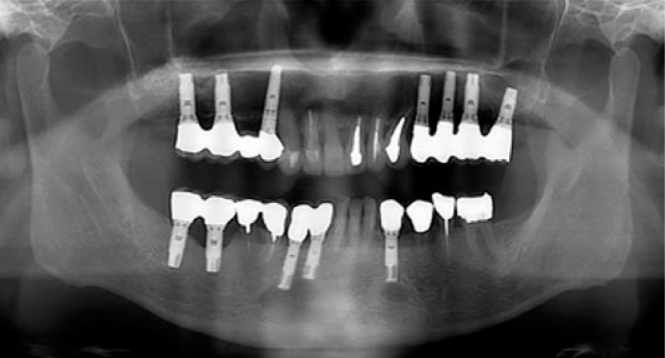
Representative orthopantogram used for the screening of patients with dental implants.

Figure 1 and Table 1 present an overview of the patient selection process and general characteristics. Among the 1685 patients referred to our hospital for oral hygiene management before malignancy therapy between April 2014 and September 2016, the presence of implants was confirmed in 49 patients by orthopantograms (Fig 2). Of these, 23 patients who underwent antitumour chemotherapy (17 men and 6 women; mean age: 63.2 ± 11.2 years; median age: 62 years) were included in our study.

**Table 1:** Overview of patients’ general characteristics

Number of patients		23
Sex	(Male/female)	17/6
Age	(Male; age: mean ± SD /median, years) (Female; age: mean ± SD /median, years)	61.2 ± 11.1/62 69.0 ± 10.3/70
Total number of dental implants	(minimum/maximum/median/total implants)	1/14/4/100
Total number of natural teeth	(minimum/maximum/median/total teeth)	7/26/19/410

The maximum, minimum, and median numbers of dental implants were 14, 1, and 4, respectively. The maximum, minimum, and median numbers of remaining natural teeth were 26, 7, and 19, respectively. The mean number of years elapsed after dental implant insertion was 4.22 ± 4.21 years (maximum: 15 years; minimum: 0 years; median: 4 years; Fig 1).

There were 9 and 14 patients in the groups with and without peri-implantitis, respectively. The affected implants included 1 maxillary anterior implant, 8 maxillary molar implants, 1 mandibular anterior implant, and 6 mandibular molar implants. Several patients developed peri-implantitis in multiple implants (Table 2). The mean number of dental implants was significantly higher in the group with peri-implantitis (5.67 ± 3.7; median: 5) than in the group without peri-implantitis (3.0 ± 3.11; median: 2; p < 0.05; Table 2). Before initiating antitumour chemotherapy, 1 patient in the peri-implantitis group and 12 in the group without peri-implantitis received dental treatment at our institute, while 8 patients in the peri-implantitis group and 2 in the group without peri-implantitis began dental treatment after the initiation of antitumour chemotherapy. The incidence of peri-implantitis was significantly higher among patients who received professional oral health care for caries treatment and/or calculus removal after initiating antitumour chemotherapy than among those who started receiving dental care before initiating chemotherapy (p < 0.001; Table 2).

**Table 2:** Parameters associated with the onset of peri-implantitis

		Group without peri-implantitis	Group with peri-implantitis	p-value
Onset of peri-implantitis	(Patients/%)	14/39.1%	9/60.9%	
Number of dental implants	(mean ± SD/median; implants)	3.50 ± 3.11/2	5.67 ± 3.67/5	0.04*
Years elapsed since the recovery of masticatory function due to implant insertion	(mean ± SD/median; years)	4.36 ± 3.78/4	4.00 ± 5.13/3	0.20
History of peri-implantitis	(Patients/%)	8/61.5	5/50.0	0.69
Number of remaining natural teeth	(mean ± SD/median)	19.21 ± 5.35/21	15.67 ± 5.92/16	0.11
Plaque control record of 20% or less	(Patients/%)	1/7.7	0/0	0.61
Patients who continued routine maintenance	(Patients/%)	7/50.0	0/0	0.01*
Initiation of dental intervention at our department	(before chemotherapy/after chemotherapy)	12/2	1/8	0.0007*

SD: standard deviation. *Statistically significantly different (p < 0.05).

The number of years elapsed since dental implant insertion was 4.0 ± 3.13 (median: 3 years) and 4.36 ± 3.78 (median: 4 years) years in the groups with and without peri-implantitis, respectively. A history of peri-implantitis at the time of initial presentation at our department was noted in 5 and 8 patients in the group with and without peri-implantitis, respectively. Peri-implantitis was observed in 1 maxillary anterior implant and 8 maxillary molar implants. There were 3 and 6 patients with peri-implantitis, depending on the time of onset, before and during chemotherapy, respectively. However, neither of these differences were significant.

The number of dental implants was significantly higher in the group with peri-implantitis. Oral hygiene condition, hygiene maintenance after implant insertion, and differences in the duration of onset of peri-implantitis are presented in Table 2 in the order of initiation of dental intervention at our department. Only 7 patients in the group without peri-implantitis had undergone continued oral hygiene maintenance before being referred to our department after dental implant insertion. Of these, only one had good oral hygiene. There were significantly fewer peri-implantitis sites in patients who had undergone continued oral hygiene maintenance after dental implant insertion than those who had not undergone continued oral hygiene maintenance (p < 0.05; Table 3).

**Table 3:** List of patients without and with peri-implantitis

	Sex	Age (years)	Tumour site/type	Regimen	Past history	Oral administration	Smoking	Initiation of dental intervention
**Patients without peri-implantitis**								
1	F	78	Oesophagus	FP	Heart disease	NAD	No	After start of chemotherapy
2	F	79	Oesophagus	FP	Heart disease	NAD	No	Before start of chemotherapy
3	M	79	Oesophagus	DCF	Hypertension, diabetes	Candesartan, Cilexetil	Yes	Before start of chemotherapy
4	M	62	Lung	CBDCA+nab-PTX	Bone metastasis, hypertension, diabetes	Gefitinib, Irbesartan, Amlodipine	Yes	Before start of chemotherapy
5	M	40	Lung	CBDCA+nab-PTX	Heart disease	Betamethasone, rrsodeoxycholic acid, Afatinib	Yes	Before start of chemotherapy
6	M	67	Pancreas	GEM+RT	Hypertension, diabetes	Amlodipine	Yes	Before start of chemotherapy
7	F	59	Hypopharynx	Post-operative CRT CDDP	None	NAD	Yes	Before start of chemotherapy
8	M	67	Hypopharynx	CDDP-RT	Hypertension, cerebrovascular disease	Amlodipine	Yes	Before start of chemotherapy
9	M	67	Oropharynx	CDDP-RT	None	NAD	Yes	Before start of chemotherapy
10	M	62	Maxillary sinus	Intraarterial injection CDDP+RT	None	NAD	Yes	Before start of chemotherapy
11	M	66	Maxillary sinus	CDDP+RT	Hypertension, diabetes	Nifedipine, Sitagliptin	Yes	Before start of chemotherapy
12	M	45	Acute myeloid leukaemia	IDR-AraC	None	NAD	Yes	After start of chemotherapy
13	M	52	Follicular lymphoma	Flu+MEL TBI BMT	Diabetes	Tacrolimus, Paroxetine	No	Before start of chemotherapy
14	M	58	Malignant lymphoma	R-CHOP	Diabetes	NAD	No	Before start of chemotherapy
**Patients with peri-implantitis**								
1	M	79	Oesophagus	DCF	Hypertension, diabetes	Amlodipine	Yes	After start of chemotherapy
2	M	72	Oesophagus	FP	Hypertension, heart disease, cerebrovascular disease, diabetes, anaemia	Bayaspirin, Amiodarone	Yes	After start of chemotherapy
3	M	62	Lung	CBDCA+nab-PTX	Bone metastasis, hypertension	Amlodipine	Yes	After start of chemotherapy
4	F	58	Hypopharynx	CRT FP	None	NAD	Yes	After start of chemotherapy
5	F	62	Glandula mammaria	PTX+Bev	Bone metastasis, lung metastasis, brain metastasis, heart disease, anaemia	Pregabalin, Morphine	No	After start of chemotherapy
6	M	45	Acute myeloid leukaemia	DNR+AraC	None	Bisoprolol fumarate	Yes	After start of chemotherapy
7	F	78	Malignant lymphoma	R-THP-COP	Lung metastasis, liver metastasis, brain metastasis, hypertension, heart disease, anaemia	Amlodipine	No	After start of chemotherapy
8	M	57	Adult T-cell lymphoma	CHOP	Brain metastasis, diabetes	Mexiletine, Lasix, Valixa	Yes	Before start of chemotherapy
9	M	59	Myelofibrosis	Flu+Bu2 TBI BMT	Anaemia	Tacrolimus, Cyclosporine	No	After start of chemotherapy

NAD: no appreciable disease; Ara-C: Cytarabine; Bev: Bevacizumab; BMT: bone marrow transplantation; CBDCA: carboplatin; CHOP: cyclophosphamide+ hydroxydaunorubicin+ vincristine+ prednisolone; CRT: chemoradiotherapy; DCF: Docetaxel+ cisplatin +5-FU; Flu: fludarabine phosphate; FP: 5-FU+ cisplatin; GEM: Gemcitabine; IDR: Idarubicin; MEL: Melphalan; nab-PTX: nanoparticle albumin–bound paclitaxel; R: Rituximab; RT: radiotherapy; TBI: total body irradiation; THP-COP: Cyclophosphamide+ vincristine+ prednisolone+ pirarubicin

**Fig 3:**
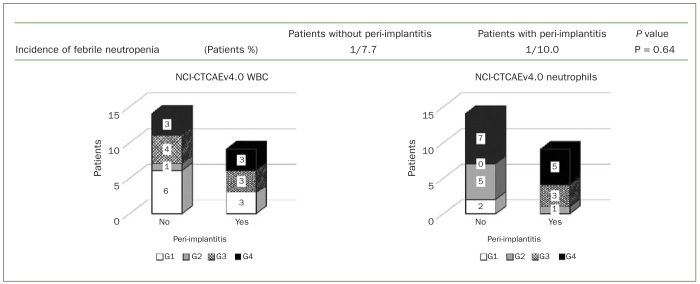
Onset of febrile neutropenia. No statistically significant difference was found in the incidence of febrile neutropenia between the peri-implantitis group and the group without peri-implantitis; however, a statistically significant difference in the neutrophil count is evident. G1, G2, etc refer to the grading system of the National Institute of Health’s Common Terminology Criteria for Adverse Events.

The CTCAE outlines a grading system from G1 to G4 for various adverse events, wherein higher grades indicate events of increased severity. The numbers of patients in each grade are presented in Fig 3. A decrease in white blood cell levels was observed in three of the G1 patients, three of the G3 patients, and three of the G4 patients in the peri-implantitis group. This decrease was also observed in six of the G1 patients, one of the G2 patients, four of the G3 patients, and three of the G4 patients in the group without peri-implantitis. Neutropenia was observed in one G2 patient, three G3 patients, and five G4 patients in the peri-implantitis group and in two G1 patients, five G2 patients, and seven G4 patients in the group without peri-implantitis. There was no statistically significant difference in any of these parameters between the two groups, and febrile neutropenia was not increased even in patients with peri-implantitis (Fig 3).

## Discussion

Implant therapy is being universally used to treat completely and partially edentulous arches and single missing teeth. However, the incidence of complications associated with implant therapy, including peri-implantitis, has also increased.^8,12^

The onset of peri-implantitis is associated with oral hygiene status, history of periodontal disease, smoking, history of peri-implantitis, and bacterial infection in the peri-implant pocket.^1-3,12,18^ According to the Consensus Report of the Sixth European Workshop on Periodontology in 2008, 28%–56% of individuals with dental implants develop peri-implantitis, and approximately 80% of individuals with dental implants develop peri-implant mucositis.^10,21^ When we investigated the implant conditions during antitumour chemotherapy in our study, we found that the incidence of peri-implantitis increased from 13.0% at the initial diagnosis to 60.9% during hospitalisation.

The protocol for collaboration between medicine and dentistry in treating malignancy is that after consultation between the oncology department and the dental department, the dental department teaches patients the importance of oral hygiene management during the treatment of malignant diseases. The dentist diagnoses the patient via an orthopantomogram and performs an oral examination to check for caries and root canal lesions. If a denture is used, the dentist checks for pressure sores on the oral mucosa, the stability and fit of the denture, and oral mucosa abnormalities, including the presence of oral cancer. However, since the priority is to treat malignant diseases, invasive dental treatment is avoided as much as possible. If a dental infection is evident, antimicrobial agents are prescribed. Next, the dental hygienist instructs the patient on brushing, removing fur coating from the tongue, and cleaning the oral mucosa. Then, the removal of tartar and mechanical cleaning of the teeth is performed. For patients with haematological malignancies and other patients who bleed easily, care should be taken not to remove tartar too far below the gingival margin. For treating peri-implantitis during chemotherapy, we provided oral care by a dental hygienist almost every weekday. Specifically, if there was pain or bleeding in the oral mucosa, we brushed with an extremely soft toothbrush and interdental brush, cleaned the pockets with 0.05% chlorhexidine gluconate, and injected 2% minocycline hydrochloride ointment into the pockets. Oral hygiene management is also continued during malignancy chemotherapy. In our study, only 1 of 13 patients who received interventions before antineoplastic agent administration had good oral hygiene at the initial visit. However, oral hygiene vastly improved upon starting chemotherapy. In contrast, all nine patients who started receiving dental treatment at our department after initiating antitumour chemotherapy showed poor plaque control (50%–100%). We provided these individuals with oral hygiene management and self-care instructions according to their systemic condition, because they presented with discomfort in the oral cavity, i.e., oral mucositis and peri-implant mucositis. However, these conditions were assessed before the onset of peri-implantitis in 2 of the 9 patients, suggesting that oral hygiene management is effective even after a patient experiences discomfort in the oral cavity. Nevertheless, the frequent consultations required to prevent severe myelosuppression after administering antitumour agents underscore the need for dental care before chemotherapy.

Fatigue, fever, malaise, nausea, and vomiting resulting from antitumour chemotherapy can lead to compromised self-care.^20^ Myelosuppression, oral mucositis, oral dryness, oral candidiasis, and poor oral hygiene increase the patient’s susceptibility to peri-implantitis by affecting the oral environment. In our study, 13 of the 23 patients showed myelosuppression (CTCAE G3 or more for white cell and neutrophil counts), and there was no statistically significant difference in the incidence of myelosuppression between the two groups. However, peri-implantitis did not cause an increase in febrile neutropenia. This suggests that with adequate professional oral hygiene management, antitumour chemotherapy can be safely administered even in the presence of peri-implantitis.

Gram-negative anaerobic bacilli can colonise peri-implantitis pockets. The tongue and oral mucosa act as reservoirs that transmit periodontopathic bacteria from the periodontal pocket to the peri-implant pocket in cases of partially edentulous arches and single missing teeth, as well as when dental implants and natural teeth coexist. This could cause peri-implantitis.^12,14-16^ The incidence of peri-implantitis increases with the number of years that have elapsed after implant insertion.^8,13,14^ However, 6 of 9 patients in our study developed peri-implantitis within 5 years after implant insertion, and 2 patients developed peri-implantitis within 1 year.

Out of 16 patients, 9 discontinued oral hygiene maintenance after implant insertion. Of these patients, 7 discontinued maintenance after the onset of malignancy. Therefore, we speculated that the diagnosis of malignancy and the associated treatment might cause patients to deprioritise the maintenance of dental implants.

Antitumour chemotherapy is often prolonged. Removal of dental implants can reduce the probability of developing peri-implantitis in patients at high risk of developing peri-implantitis, because osseointegration is inhibited by the inflammatory reaction associated with oral bacteria.^4,5,15^ Therefore, cooperation between the departments of oncology and dentistry is vital in such cases. If the removal of dental implants is difficult, the superstructure and abutment can be temporarily removed while implants remain submerged under the subperiosteal and gingival margins to render plaque control relatively easy.

In our study, 2.9% of patients had dental implants (49/1685 patients), and 46.9% of these patients underwent antitumour chemotherapy (23/49 patients). Oral hygiene management before malignancy chemotherapy was effective in preventing peri-implantitis. This intervention might serve as a prophylactic procedure against febrile neutropenia precipitated by peri-implantitis, even when initiated during chemotherapy. However, this study has some limitations. First, it had a relatively small sample size. In addition, because it was a single-centre study, a certain amount of selection bias was inevitable. Further, we were unable to evaluate causality. All limitations and potentials of bias inherent to the retrospective study design apply to this study as well. Finally, the results cannot be generalised to different populations.

## Conclusion

In patients with dental implants who undergo malignancy chemotherapy, oral hygiene management before malignancy chemotherapy is essential if myelosuppression is expected. This appears to be particularly true for patients with multiple dental implants. This study suggested that management of oral hygiene through early and continued cooperation between the departments of oncology and dentistry is necessary in patients with dental implants requiring antitumour chemotherapy.
